# Unrestricted somatic stem cells from human umbilical cord blood grow in serum-free medium as spheres

**DOI:** 10.1186/1472-6750-9-101

**Published:** 2009-12-15

**Authors:** Faten Zaibak, Paul Bello, Jennifer Kozlovski, Duncan Crombie, Haozhi Ang, Mirella Dottori, Robert Williamson

**Affiliations:** 1 Department of Paediatrics, The University of Melbourne, Royal Children's Hospital, Victoria, 3052, Australia; 2 Murdoch Childrens Research Institute, Royal Children's Hospital, Victoria, 3052, Australia; 3 Stem Cell Sciences Ltd, Babraham Research Campus, Cambridge, CB22 3AT, UK; 4 O'Brien Institute, Melbourne, Victoria, 3065, Australia; 5 Centre for Neurosciences, Department of Pharmacology, The University of Melbourne, Parkville, Victoria, 3010, Australia

## Abstract

**Background:**

Human umbilical cord blood-derived unrestricted somatic stem cells (USSCs), which are capable of multilineage differentiation, are currently under investigation for a number of therapeutic applications. A major obstacle to their clinical use is the fact that *in vitro *expansion is still dependent upon fetal calf serum, which could be a source of pathogens. In this study, we investigate the capacity of three different stem cell culture media to support USSCs in serum-free conditions; HEScGRO™, PSM and USSC growth medium^ACF^. Our findings demonstrate that USSCs do not grow in HEScGRO™ or PSM, but we were able to isolate, proliferate and maintain multipotency of three USSC lines in USSC growth medium^ACF^.

**Results:**

For the first one to three passages, cells grown in USSC growth medium^ACF ^proliferate and maintain their morphology, but with continued passaging the cells form spherical cell aggregates. Upon dissociation of spheres, cells continue to grow in suspension and form new spheres. Dissociated cells can also revert to monolayer growth when cultured on extracellular matrix support (fibronectin or gelatin), or in medium containing fetal calf serum. Analysis of markers associated with pluripotency (*Oct4 and Sox2*) and differentiation (*FoxA2, Brachyury, Goosecoid, Nestin, Pax6, Gata6 and Cytokeratin 8*) confirms that cells in the spheres maintain their gene expression profile. The cells in the spheres also retain the ability to differentiate *in vitro *to form cells representative of the three germline layers after five passages.

**Conclusions:**

These data suggest that USSC growth medium^ACF ^maintains USSCs in an undifferentiated state and supports growth in suspension. This is the first demonstration that USSCs can grow in a serum- and animal component-free medium and that USSCs can form spheres.

## Background

Human umbilical cord blood contains a subset of stem cells that can differentiate into cells representative of all three germline layers [1-4]. We follow Kögler, the first to describe the multilineage capacity of these cells *in vivo*, in calling them "unrestricted somatic stem cells" (USSCs) [1]. A number of studies demonstrate the therapeutic potential of USSCs in bone healing, as accessory cells to reduce graft-*versus*-host disease, in the repair of myocardial infarcts and as vehicles for gene therapy [1,3,5-8].

Although USSCs are rare compared to haematopoietic stem cells in cord blood, they can be expanded rapidly to yield large numbers of cells for study or transplantation. Culturing of USSCs depends on growth in medium containing a high concentration of specific batches of fetal calf serum. When cultured in fetal calf serum, USSCs form an adherent monolayer, do not require a feeder layer, and can be cultured without differentiating or losing their proliferative capacity.

Before USSCs can be used therapeutically, a method for large scale amplification of cells under Good Manufacturing Practice (GMP) conditions is necessary, preferably using defined medium free of xenobiotic components. Fetal calf serum contains undefined factors, which may vary from batch to batch, and that may activate or inhibit stem cell differentiation. The growth of cells in defined medium not only reduces variability, but also eliminates the need for costly and time consuming fetal calf serum batch testing. Additionally, medium that is free of non-human products reduces concerns about transmission of infectious cross-species pathogens (such as prions or zoonosis), and possible immunogenic responses to non-human proteins [9-11].

Here we investigate the capacity of three different stem cell culture media HEScGRO™, PSM and USSC growth medium^ACF ^to enable maintenance of USSCs in serum-free conditions.

PSM is of interest as it contains Sphingosine-1-phosphate (S1P) and platelet-derived growth factor (PDGF), factors known to enable the survival and proliferation of human embryonic stem (hES) cells in an undifferentiated state for a prolonged period of time [12,13].

S1P is a bioactive sphingolipid present in serum that binds to its own G protein-coupled receptors, sphingosine-1-phosphate receptor 1 to 5 (S1P_(1-5)_), and activates various intracellular signaling pathways depending on the cell type [14-18]. S1P regulates key biological processes such as cell proliferation, motility, migration and survival in both adult and embryonic stem cell types [13,19-21].

PDGF is a growth factor, also present in serum, that is widely described as a potent mitogen [22]. PDGF is comprised of homo- and hetero-dimeric isoforms of polypeptide chains A, B, C and D [23,24]. The response of a cell to a given isoform of PDGF is dependent on the signaling pathways activated by the two platelet-derived growth factor receptors (PDGFR-α and PDGFR-β), and the capacity of the cell to respond to these signals [25-27].

HEScGRO™ is a commercial serum- and animal component-free, defined medium known to support the growth and pluripotency maintenance of hES cells. The media contains FGF2 (basic FGF) that, like PDGF, has been reported as having proliferative and differentiation effects on various cell types [28], and figures quite prominently in numerous other adult [29], or embryonic-derived stem cell media formulations [30].

## Results

### USSCs form spheres in USSC growth medium^ACF^

**Figure 1:**
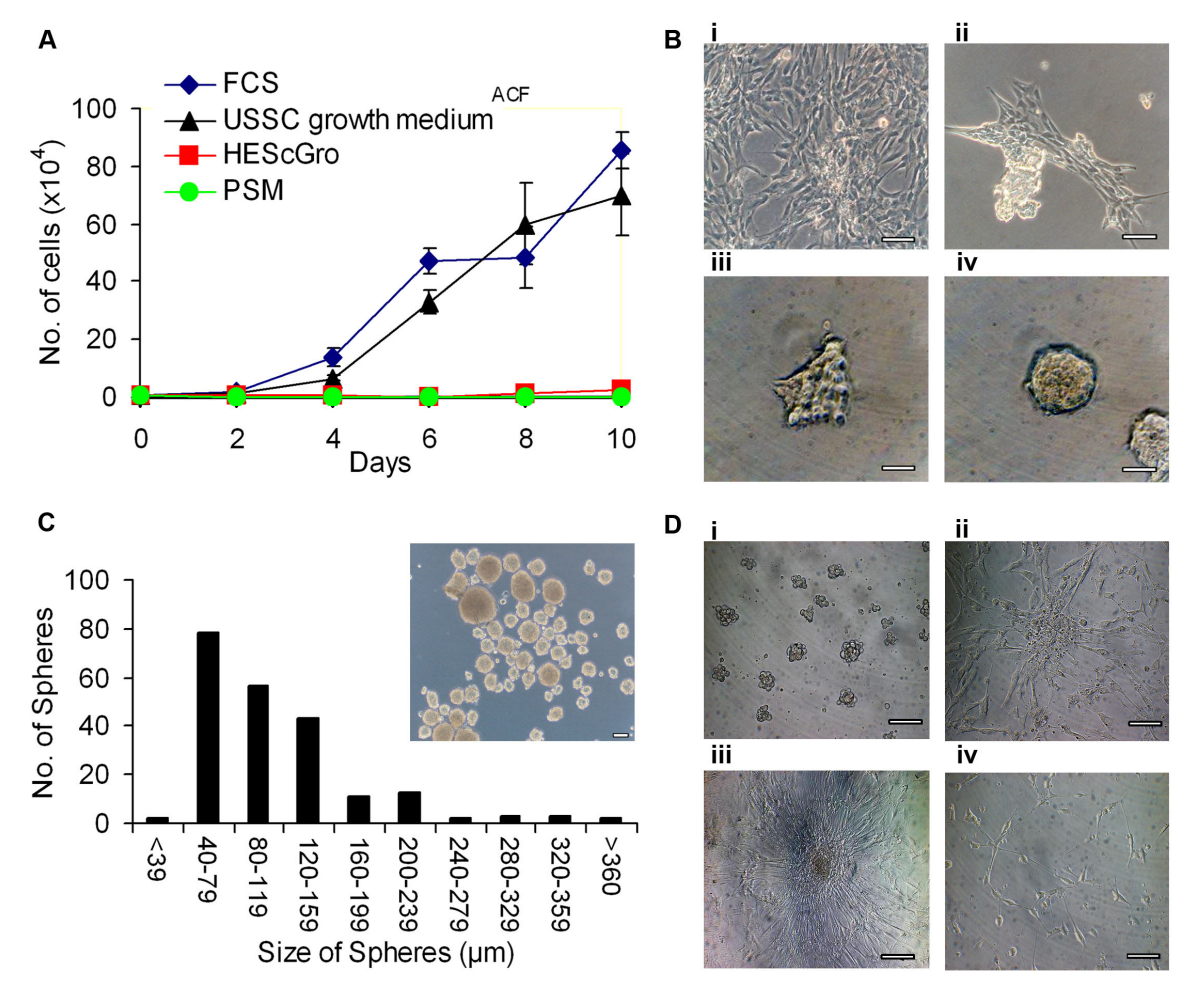
**Derivation and maintenance of spheres**. **A**, Growth kinetics of USSCs cultured in stem cell proliferation medium, HEScGRO and PSM and USSC growth medium^ACF^. Cells were seeded at low density and total cell number was determined every two days. **B**, Time course of the formation of spheres. **i**, USSCs in stem cell proliferation medium; **ii**-**iv**, USSCs in USSC growth medium^ACF^; **ii**, after three passages USSCs begin to cluster; **iii**, within 24 hours of formation, clusters detach from the surface; **iv**, with continued culturing, clusters increase in size and have defined boundaries. (Magnification, ×200; scale bar is 100 μm)**C**, Size of spheres on day seven. (Magnification, ×100; scale bar is 100 μm)**D**, Growth of spheres after mechanical dissociation and re-plating on extracellular matrix-coated and non-ECM-coated surfaces; **i**, clusters of cells continue to grow in USSC growth medium^ACF^; **ii**, dissociated spheres can grow as an adherent monolayer on fibronectin and collagen-coated surfaces in USSC growth medium^ACF^; **iii**, dissociated spheres can grown as an adherent monolayer on gelatin-coated surfaces in USSC growth medium^ACF^; **iv**, in the absence of mechanical dissociation, spheres cultured in stem cell proliferation media grow as an adherent monolayer. (Magnification, ×200; scale bar is 100 μm).

We have analyzed the growth of USSCs in three serum-free media formulations; HEScGRO, PSM and USSC growth medium^ACF^. Figures 1A clearly demonstrate that cells cultured in HEScGRO and PSM do not grow, whereas cells grown in USSC growth medium^ACF ^proliferate at the same rate as cells grown in stem cell proliferation medium containing 30% fetal calf serum.

However, after one to three passages in USSC growth medium^ACF ^cells start to form small clusters, which become tight spheres after a further 24 hours (Figure 1B). These spheres are initially heterogeneous in shape, but with continued culturing, rounded spheres with defined borders are observed. Seven days after their first appearance, the size of the spheres varies. Figure 1C shows that the majority of spheres (64%) are small in diameter (<120 μm), 31% are medium (120-240 μm) and the remaining spheres (5%) are large (>240 μm). The smallest sphere observed was 30 μm, and the largest was 475 μm.

Figure 1D shows that the spheres can be mechanically dissociated and can reform spheres upon culturing in USSC growth medium^ACF^. Evidence of proliferation was observed as spheres could be passaged and new spheres were observed to form.

**Figure 2:**
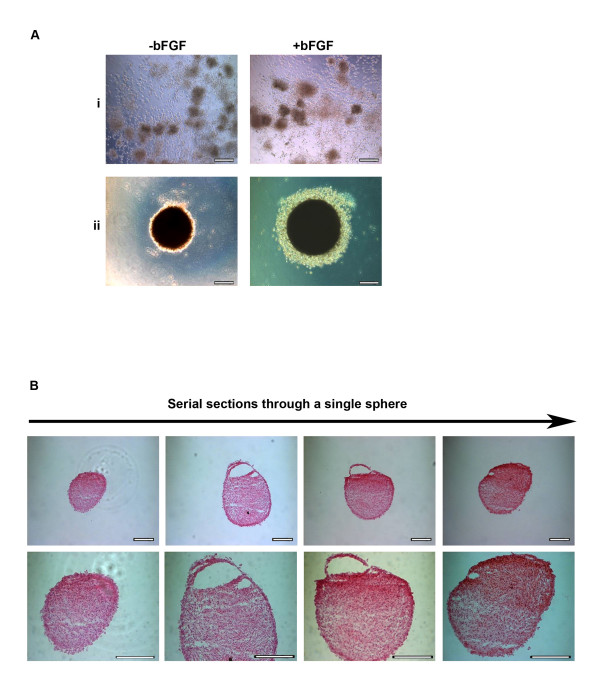
**Characterisation of spheres**. **A**, Morphology of spheres seven days after seeding at high density (1 × 10^4 ^cells/30 μl) as hanging drops in the presence or absence of bFGF in: **i**, stem cell proliferation medium without fetal calf serum; or **ii**, USSC growth medium^ACF^. (Magnification, ×200; scale bar is 100 μm) **B**, Serial sections of spheres stained with haematoxylin and eosin, generated by culturing in USSC growth medium^ACF ^for seven days at both ×200 and ×400 magnification; scale bar is 100 μm.

As the culturing of stem cells as spheres is important for a number of applications, we set out to confirm whether USSCs could form spheres using one of the standard sphere formation methods [31,32]. Using the serum-free hanging drop method, we cultured cells either in USSC growth medium^ACF^, or stem cell proliferation media without fetal calf serum. Figure 2A shows that cells grown in stem cell proliferation media without fetal calf serum in the presence or absence of bFGF do not form spheres, but cells grown in USSC growth medium^ACF ^always form spheres. There also appears to be a differential effect on the morphology of the sphere depending on whether bFGF is used, in that spheres derived using bFGF are smaller, but tighter, and have less of a halo.

To determine their structure, spheres were serially sectioned and stained with haematoxylin and eosin. Figure 2B shows representative images of a serial section of a sphere. The cells in the spheres appear homogenous in size and morphology and 48% of the spheres also contained cavities.

Altogether, these data suggest that USSC growth medium^ACF ^enables USSC proliferation, that this growth occurs simultaneously with the loss of the cells' ability to adhere to plastic, and that these non-adherent cells can continue to grow in suspension as spheres.

### USSC-derived spheres can revert to monolayer growth

The ability of mechanically dissociated spheres to revert to monolayer growth and continue to proliferate was studied after culture in USSC growth medium^ACF ^on fibronectin or gelatin coated surfaces, or in the presence of stem cell proliferation media containing fetal calf serum. Figure 1D shows that the cells from the spheres re-adhere and morphologically resemble the parental USSCs in the presence of fetal calf serum, or when the surface is coated with extracellular matrix proteins. These cells were cultured for up to two weeks, during which they were passaged three times and expanded up to six-fold. This confirms that cells within the spheres, which have been cultured in USSC growth medium^ACF^, can revert to monolayer growth and continue to proliferate.

### USSCs maintain their multipotentiality when cultured as spheres

**Figure 3:**
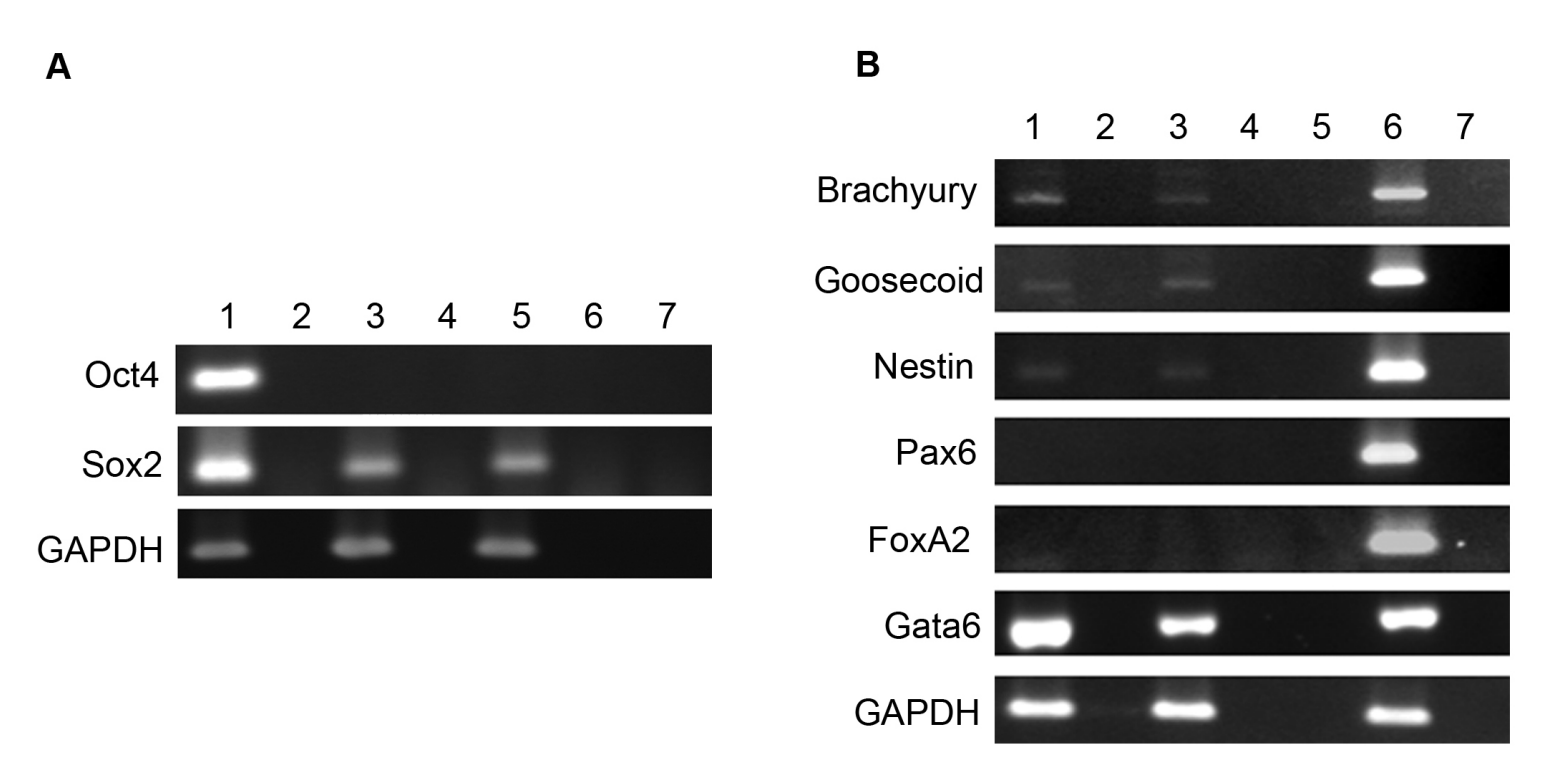
**Gene expression profile seven days after sphere formation**. **A**, RT-PCR for pluripotency-associated genes. RT-PCR shows an absence of *Oct4 *gene expression in both USSCs and seven day spheres, yet demonstrates expression of the another pluripotency-associated marker *Sox2*. Lane 1, hES cells; Lane 2, RT-negative control of the hES cells; Lane 3, USSCs; Lane 4, RT-negative control of the USSCs; Lane 5, day seven spheres; Lane 6, RT-negative control of the day seven spheres; and Lane 7, water control. **B**, RT-PCR of genes representative of the three germline layers show that day seven spheres maintain the same expression profile as adherent USSCs. Lane 1, USSCs; Lane 2, RT-negative of the USSCs; Lane 3, day seven spheres; Lane 4, RT-negative for the day seven spheres; Lane 5, water control; Lane 6, positive control, spontaneously differentiated hES cells; Lane 7, RT-negative of the spontaneously differentiated hES cells. The house keeping gene GAPDH was used as a positive control.

**Figure 4:**
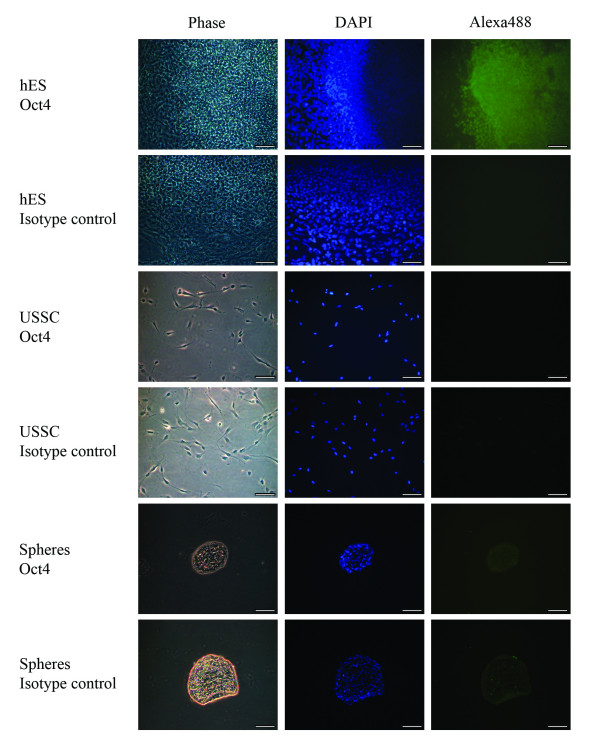
**Oct4 staining on USSC-derived spheres**. Representative microscopy images of seven day spheres fixed and stained with an Oct4 antibody (Alexa488; green) and counter stained with 4, 6-diamidino-2-phenylindole (DAPI; blue). As a positive control, the nuclear localisation of the Oct4 antibody was confirmed in hES cells. USSCs were also probed to confirm the Oct4 status of the starting cell population. The respective negative isotype control images are also shown. USSCs and seven day spheres do not express Oct4 at the protein level. (Magnification, ×200; scale bar is 100 μm).

To determine if day seven spheres express pluripotency-associated markers, the expression of *Oct4 *and *Sox2 *was determined by RT-PCR. Figure 3 shows that USSCs grown in adherent culture do not express *Oct4*, but do express *Sox2*, and that USSCs grown as spheres maintain this expression profile. In view of recent reports questioning accurate detection of *Oct4 *mRNA expression [33], we confirmed that the primers used in this study do not bind to *Oct4B *(isoform 2) and are specific to the cDNA of the nuclear-localized *Oct4A *(isoform 1). We also confirmed this result using antibody staining. The antibody staining clearly detects nuclear-localized Oct4 in hES cells, but expression could not be detected in USSCs nor seven day spheres (Figure 4).

**Figure 5:**
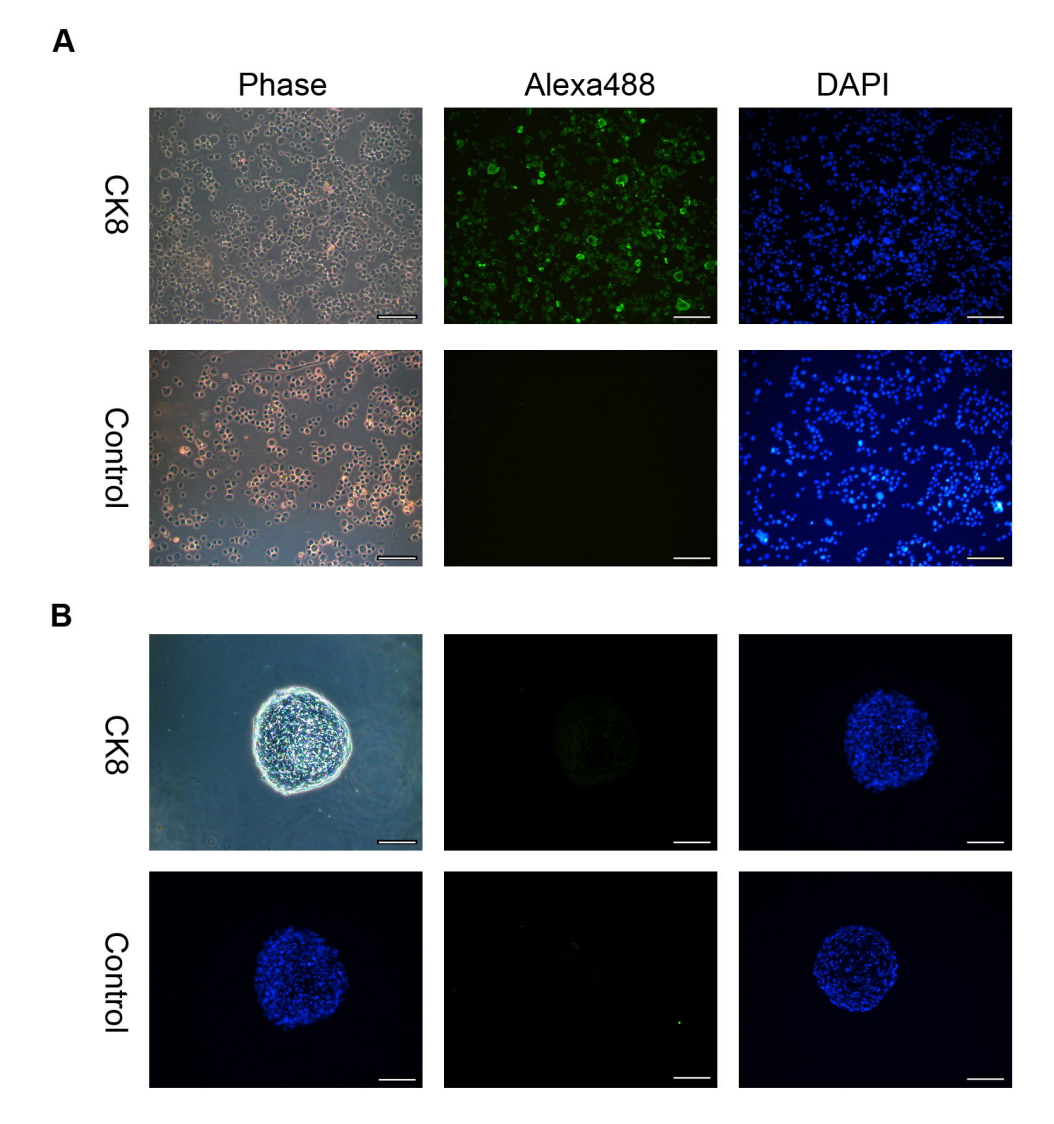
**Cytokeratin 8 staining on USSC-derived spheres**. **A**, As a positive control for CK8 expression, primary human bronchial epithelial (16HBE14o-) cells were stained. **B**, Representative microscopy images of seven day spheres fixed, sectioned and stained with a Cytokeratin 8 (CK8) antibody (Alexa488; green) and counter stained with 4, 6-diamidino-2-phenylindole (DAPI; blue). The respective negative isotype control images are also shown. (Magnification ×200; scale bar is 100 μm.)

**Figure 6:**
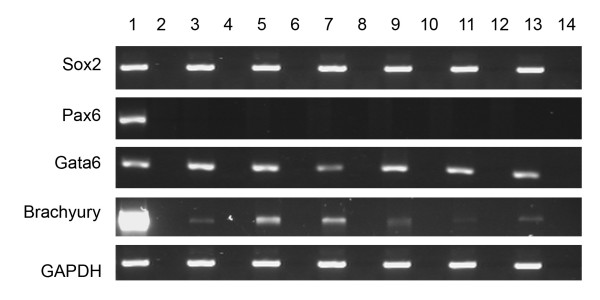
**Gene expression profile of three different stem cell lines after five passages in USSC growth medium^ACF^**. RT-PCR for pluripotency (*Sox2*), ectodermal (*Pax6*), mesodermal (*Gata6*) and endodermal (*Brachyury*), associated genes after five passages in USSC growth medium^ACF^. The house keeping gene *GAPDH *was used as a positive control. Lane 1, hES cells; Lane 2, RT-negative control for hES cells. Lanes 3-8 are USSCs cultured in fetal calf serum: Lane 3, USSC Line 1; Lane 4, RT-negative control for USSC Line 1; Lane 5, USSC Line 2; Lane 6, RT-negative control for USSC Line 2; Lane 7, USSC Line 3; and Lane 8, RT-negative control for USSC Line 3. Lanes 9-14 are USSCs passaged five times in USSC growth medium^ACF^: Lane 9, USSC Line 1; Lane 10, RT-negative control for USSC Line 1; Lane 11, USSC Line 2; Lane 12, RT-negative control for USSC Line 2; Lane 13, USSC Line 3; and Lane 14, RT-negative control for USSC Line 3.

To determine if the cells in the spheres initiate a differentiation program, we analysed markers associated with commitment of stem cells. Figures 3 and 5 show there is no change in the expression profile of markers associated with ectodermal (*Pax6 *and *Nestin*), mesodermal (*Brachyury and Goosecoid*) and endodermal (*FoxA2*, *Cytokeratin 8 *and *Gata6*) differentiation in day seven spheres when compared to USSCs. This expression profile is also maintained after five passages as determined by RT-PCR for Sox2, Pax6, Gata6, and Brachyury (Figure 6).

### Spheres can be differentiated to show expression of markers representative of the three germline layers

**Figure 7:**
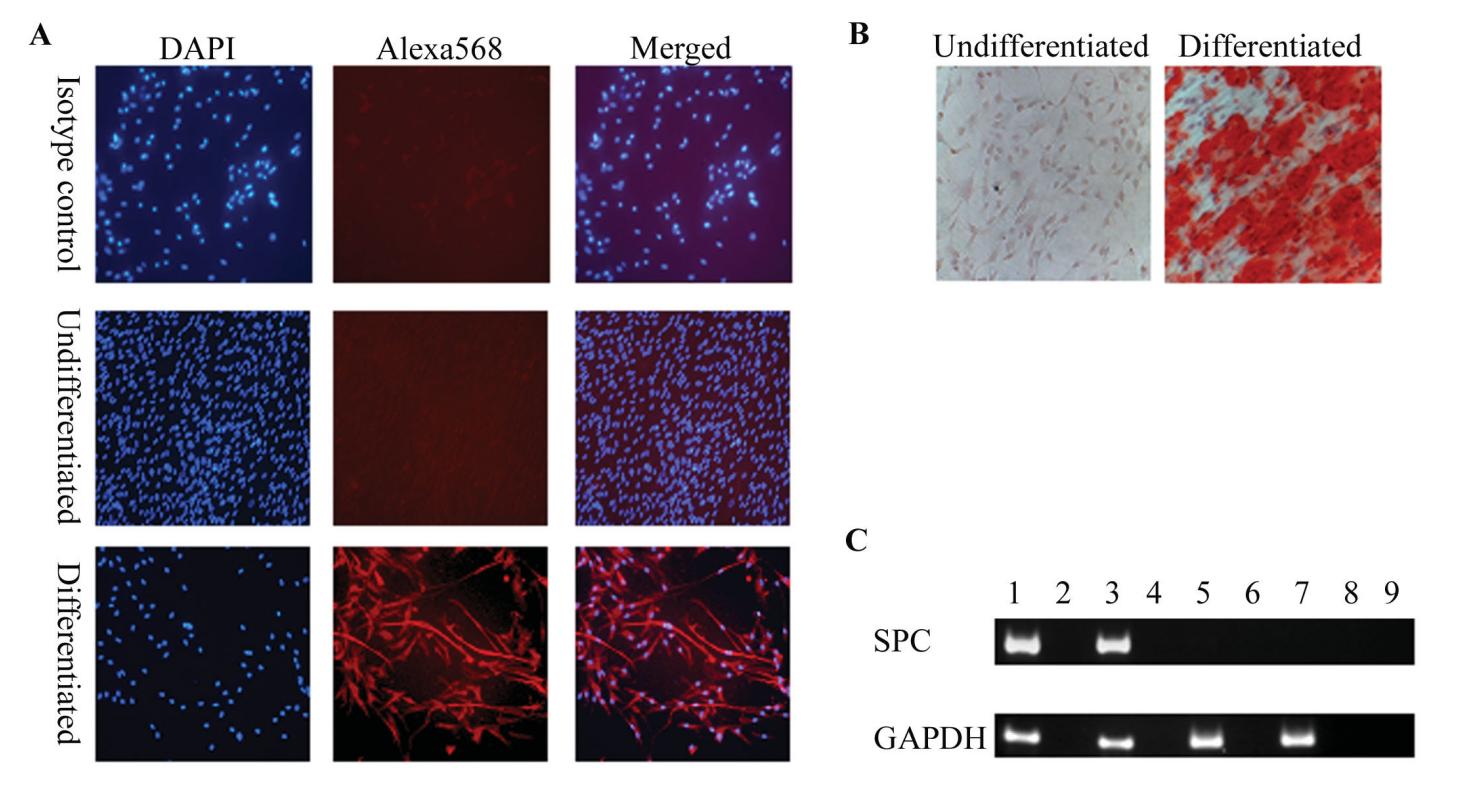
**Differentiation of spheres into neuronal, bone and epithelial-like cells**. Seven days after sphere formation, cells were dissociated and plated on coated plates, as described in the materials and methods. The next day, cells were cultured in differentiation medium. **A**, Neuronal differentiation was confirmed by staining with a neuronal-specific β-tubulin III antibody (Alexa568; red) and counter stained with 4, 6-diamidino-2-phenylindole (DAPI; blue). **B**, Bone differentiation cultures were stained with Alizarin Red S to test for mineral deposition. **C**, Epithelial differentiation was confirmed by RT-PCR of SPC. Lane 1, human bronchial epithelial cell line, 16HBE14o-; Lane 2, RT-negative control of the human bronchial epithelial cell line; Lane 3, differentiated spheres; Lane 4, RT-negative control of the differentiated spheres; Lane 5, undifferentiated spheres; Lane 6, RT-negative control of the undifferentiated spheres; Lane 7, USSC Line 1; Lane 8, RT-negative control of the USSC Line 1; and Lane 9, water control.

**Figure 8:**
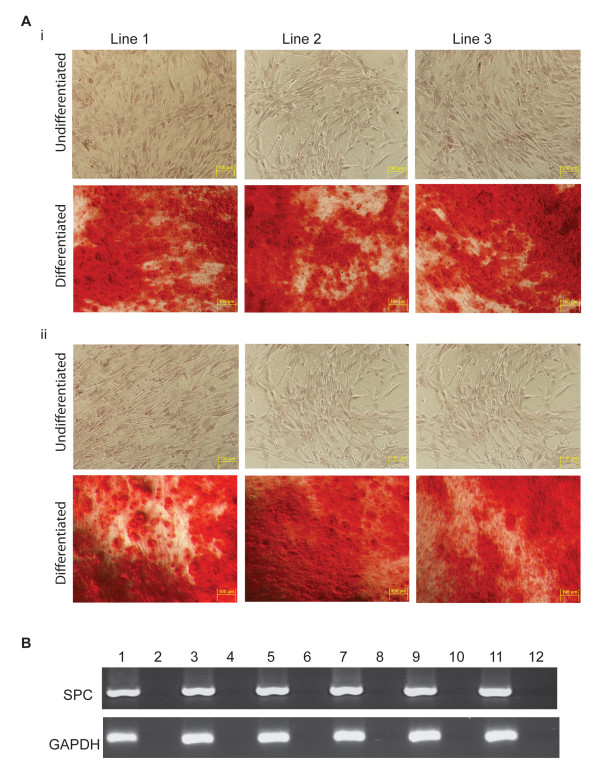
**Differentiation capacity of three different stem cell lines after five passages in USSC growth medium^ACF^**. After five passages in USSC growth medium^ACF^, cells were dissociated and plated on coated plates, as described in Materials and Methods. The next day, differentiation medium was added. **A**, Bone differentiation cultures were stained with Alizarin Red S to test for mineral deposition. (**i**) USSC cultured in fetal calf serum; and (**ii**) cells cultured in USSC growth medium^ACF ^for five passages. **B**, Epithelial differentiation was confirmed by RT-PCR of SPC of cells differentiated in Small Airway Growth Medium. Lane 1-6 USSCs passaged in fetal calf serum: Lane 1, USSC Line 1; Lane 2, RT-negative control for USSC Line 1; Lane 3, USSC Line 2; Lane 4, RT-negative control for USSC Line 2; Lane 5, USSC Line 3; and Lane 6, RT-negative control for USSC Line 3. Lanes 7-12 USSCs passaged five times in USSC growth medium^ACF^: Lane 7, USSC Line 1; Lane 8, RT-negative control for USSC Line 1; Lane 9, USSC Line 2; Lane 10, RT-negative control for USSC Line 2; Lane 11, USSC Line 3; and Lane 12, RT-negative control for USSC Line 3.

**Figure 9:**
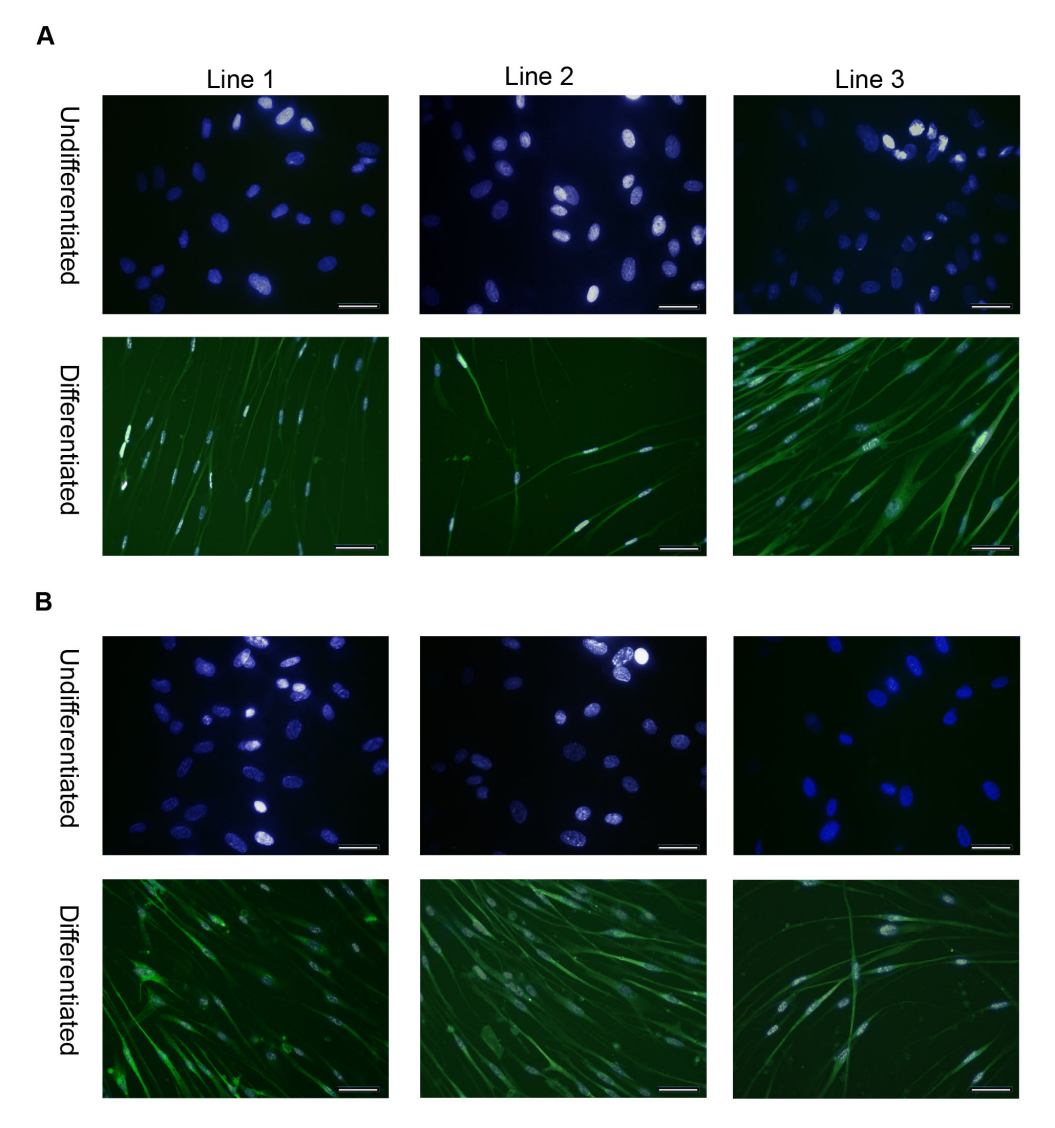
**Neuronal differentiation capacity of three different stem cell lines after five passages in USSC growth medium^ACF^**. Neuronal differentiation was confirmed by staining with a neuronal-specific β-tubulin III antibody (Alexa488). **A**, USSCs cultured in fetal calf serum; and **B**, USSCs cultured in USSC growth medium^ACF ^for five passages. DAPI (blue) and Alexa488 (green) merged images shown. (Magnification ×200; scale bar is 100 μm).

To confirm that the differentiation capacity of the cells in the spheres had been maintained; seven day spheres were dissociated and incubated in lineage-specific differentiation medium. Figure 7 shows that the cells within the spheres maintain the ability to differentiate and express markers representative of the three germline layers. Cells expressing neuronal-specific β-tubulin III (a marker of ectodermal cells), that stain positive for mineralization as detected by Alizarin Red S staining (a marker of mesodermal cells) and express epithelial-specific surfactant protein-C (a marker of endodermal cells) were all observed. The differentiation capacity of the cells is also maintained after five passages in USSC growth medium^ACF ^(Figures 8 and 9).

## Discussion

With the goal of deriving clinically safe USSCs, we aimed to culture established USSCs in the serum- and animal component-free medium, USSC growth medium^ACF^. We observe that USSCs continue to proliferate in USSC growth medium^ACF^, but after one to three passages, the cells aggregate and grow in suspension as spheres. We show that spheres can be dissociated and can continue to grow for five passages, as long as they are dissociated before the sphere becomes cystic. We also show the spheres can revert to monolayer growth when provided with extracellular matrix support or when plated in medium containing fetal calf serum. Cells passaged in USCC growth medium^ACF ^maintained their gene expression profile as judged by Sox2, Brachyury, Pax6 and Gata6 expression. Cells also maintained their capacity to form bone-like Alizarin red positive cells, SPC positive epithelial cells and β-tubulin III positive neuronal cells after directed differentiation. This is the first report of a serum-free medium that enables proliferation of USSCs and the first to report that USSCs can be grown in suspension as spheres, without losing their differentiation capacity.

Growing stem cells as spheres has been the initial step in a wide range of differentiation studies, as well as studies in pharmacogenetics, teratogenesis and tumourigenesis [32,34-42]. Sphere formation can be facilitated by growing cells in specific medium (both serum-free and complete), on non-adherent plates, in bioreactors, in agar, as hanging drops, or by centrifugation [31,36,43]. Despite these many studies, the significance of sphere formation is yet to be fully understood. While it could reflect a non-specific event, it could alternatively be an active process essential for continued development and differentiation. The differentiation potential of stem cells in spheres appears to be dependent upon the starting cell population and the medium in which the spheres are cultured. For example, human bone marrow stromal cells can be conditioned to give rise to neurospheres, which lose their stem cell characteristics and become committed to neuronal development [40].

To characterize the spheres derived in this study further, we investigated a number of markers expressed in pluripotent cells and in cells representative of the three germline layers. We show that *Oct4*-negative USSCs give rise to spheres, and that cells within these spheres continue to express *Sox2*. Analysis of differentiation-associated markers (*Nestin*, *Pax6*, *Brachyury, Goosecoid, FoxA2 *and *Gata6*) by RT-PCR confirms that cells cultured in USSC growth medium^ACF ^do not initiate a program of differentiation.

The ability of USSC spheres to maintain an undifferentiated state makes them distinct from other spheres derived from cells capable of multilineage differentiation. The most studied of these spheres are embryoid bodies, defined as spherical clusters of both pluripotent and committed stem cells that can organize in a developmental-specific manner [35,42,44-48]. Unlike embryoid bodies, USSC spheres do not commit to any differentiation programs when cultured in USSC growth medium^ACF^.

The cavities detected in USSC spheres are also distinct from cavities observed in embryoid bodies. The formation of cavities in embryoid bodies is an active process, characterized by a thickening of the wall around the cavity, and associated with the expression of endodermal-specific markers such as Cytokeratin 8 [49,50]. While USSC spheres can have cavities, we show that the cells surrounding the cavities and the outer layer of the sphere do not express the endodermal marker Cytokeratin 8.

As marker analysis is insufficient to infer function, we also analyzed the ability of the cells to respond to differentiation cues. When USSC spheres are reverted to monolayer growth and differentiated in lineage-specific medium, surfactant protein C-positive epithelial-like cells, β-tubulin III-positive neuronal-like cells and Alizarin Red S-positive bone-like cells are observed after one to five passages, confirming that the cells in the spheres are still multipotent. However, derivation of spheres from a single cell and validation that each cell in the sphere retains these stem cell properties is still essential.

## Conclusions

We present a new method to grow USSCs in suspension without losing their multi-lineage differentiation capacity in short term assays. The ability to culture these cells in a three-dimensional spherical structure in a defined animal component-free environment is a first step towards improving protocols for tissue engineering, and expansion of these cells *in vitro*.

Although there are a number of serum-free media marketed for culturing other types of stem cells, USSC growth medium^ACF ^is unique because it enables the short term expansion of USSCs in suspension, the formation of spheres, and the maintenance of the multilineage differentiation capacity of USSCs. Suspension culturing of cells facilitates large scale expansion, but we note that it will still be necessary to validate the genetic stability and safety of cells after long term cell growth before they can be used therapeutically. The latter is also heightened by two recent publications using human cord to derive iPSC [51,52] which would further expand USSC multipotentiality for therapeutic utility, including drug discovery.

## Methods

### Isolation and culture of USSCs

Cord blood was collected with informed consent from mothers undergoing elective Caesarean section. The protocol was approved by the University of Melbourne and the Royal Women's Hospital Human Ethics Review Committees. After the delivery of the baby, the cord was clamped and the blood was collected from the umbilical cord vein in bags containing 21 ml citrate phosphate dextrose (Macopharma, Mouvaux, France).

The USSC lines were successfully generated as described by Kögler *et al*. [1]. Briefly, the mononuclear cell fraction was separated on a Ficoll gradient (density 1.077 g/cm^3^, GE Healthcare, Uppsala, Sweden) at 400 g for 25 min followed by lysis of erythrocytes in ammonium chloride buffer (0.14 M ammonium chloride, 0.25 mM ethylene-diamine-tetra-acetic acid (EDTA), 10 mM *tris*(hydroxymethyl)aminomethane pH7.4) and two washing steps in calcium- and magnesium-free phosphate-buffered saline (PBS), pH 7.4. To initiate the growth of the adherent USSC colonies, the mononuclear cell suspension was plated at 2 × 10^6 ^cells/cm^2 ^in initiation medium containing low-glucose Dulbecco's Modified Eagle's Medium (Lonza, Walkersville, MD, USA) supplemented with 30% fetal calf serum (Hyclone, Logan, UT, USA), 10^-7 ^M dexamethasone (Sigma, St Louis, MO, USA), 100 U/ml penicillin (Invitrogen, Chadsworth, CA, USA), 0.1 mg/ml streptomycin (Invitrogen) and 2 mM Ultraglutamine (Lonza).

Cells were incubated at 37°C in 5% CO_2 _in a humidified incubator. Every five days, the non-adherent fraction was removed and fresh medium was added. Initiation medium was used until the first colonies appeared or for a maximum of three weeks. Expansion of the cells was carried out using initiation medium without dexamethasone (stem cell proliferation medium). After reaching 80% confluency, the cells were subcultured using 0.25% trypsin/EDTA (Invitrogen), and replated at a 1:2 dilution in stem cell proliferation medium. Low passage cells were frozen down at 1 × 10^6 ^cells/ml in 10% dimethyl sulfoxide (Sigma) and 50% fetal calf serum (Hyclone). For each experiment, cells were thawed and used before they reached passage ten.

The phenotype of the lines was as previously published [1,8]. All the lines were negative for CD34, CD24, CD31, CD45 and MHCII, and positive for CD44, CD71 and MHCI. The multilineage capacity of the lines was confirmed by differentiation into cells representative of the three germline layers.

### Culturing in serum-free medium

Cells were cultured in three different media; PSM, HEScGRO™ or USSC growth medium^ACF^. PSM; is a basal medium containing low-glucose Dulbecco's Modified Eagle Medium (Cambrex BioScience), 100 U/ml penicillin/0.1 mg/ml streptomycin (Invitrogen), and 2 mM Ultraglutamine (Cambrex BioScience) with or without 10 μM of S1P (Biomol, Exeter, UK) and 20 ng/ml PDGF (Peprotech, NJ, USA) as described in Pebay *et al*., 2007. HEScGRO™ (Millipore) was prepared according to the manufactures instructions. USSC growth medium^ACF ^(Stem Cell Sciences, UK) was supplemented with 10 ng/ml recombinant human basic fibroblast growth factor (bFGF) (Millipore) before use.

Cells were seeded at 5.2 × 10^4 ^cells/cm^2 ^and split 1:2 when they reached 80% confluency. However, after one to three passages, the cells cultured in USSC growth medium^ACF ^grew as non-adherent spheres without requiring any intervention to initiate aggregation. To maintain sphere proliferation, spheres were dissociated mechanically or using 0.25% trypsin/EDTA every four days, and allowed to reform in USSC growth medium^ACF^. Cells were cultured for five passages and then characterized further. If spheres are not dissociated within a week they become cystic and resistant to disaggregation by trypsinization.

Spheres were also generated using the hanging drop formation [31]. Briefly, a total of 1 × 10^4 ^cells were placed in 30 μl of USSC growth medium^ACF ^(plus or minus bFGF), or in stem cell proliferation medium without fetal calf serum (plus or minus bFGF), and dropped onto the inside of a Petri dish lid. Drops were incubated upside down in a Petri dish that contained PBS in the base to prevent evaporation.

To confirm the cells could revert to monolayer growth, dissociated and non-dissociated spheres were grown in stem cell proliferation medium that contains fetal calf serum. To determine if the cells could adhere while cultured in USSC growth medium^ACF^, dissociated and non-dissociated spheres were plated in Petri dishes coated with 1% gelatin (Sigma) in PBS, or 10 μg/ml fibronectin (Becton Dickinson, Franklin Lakes, NJ, USA).

### Differentiation assays

Multipotency of the USSCs and the spheres was confirmed by differentiation into bone-, neuronal- and epithelial-like cells. Spheres were dissociated with 0.25% trypsin/EDTA (Invitrogen) and plated on 0.1% gelatin (Sigma) in PBS-coated plates overnight before induction.

To test for mesodermal differentiation capacity of the cells, bone differentiation assays were performed. Cells were plated at 7 × 10^3 ^cells/cm^2 ^cultured for ten days in stem cell proliferation medium containing 10^-7 ^M dexamethasone (Sigma), 10 mM glycerol-6-phosphate (Sigma) and 50 μg/ml ascorbic acid (Sigma) as described by Jaiswal *et al. *[53]. Medium was replaced every five days. To confirm successful mineralisation, cells were fixed for ten minutes with 70% ethanol at 4°C, and stained for ten minutes with 1% Alizarin Red S (Sigma) in distilled water, pH 4.2. The cells were then washed five times in distilled water, layered in PBS (minus calcium and magnesium) and phase images were captured as described below.

To demonstrate the neuronal differentiation capacity of the cells, the differentiation protocol described by Dottori *et al. *was adapted for USSCs [54]. Briefly, cells were plated on 10 μg/ml poly-D-lysine (Sigma), 10 μg/ml fibronectin (Becton Dickinson) coated glass coverslips and cultured in neural basal medium consisting of Neurobasal A with 2% B-27, 1% insulin-transferrin-selenium-A, 1% N2, 2 mM L-glutamine, 100 U/ml penicillin and 0.1 mg/ml streptomycin (all sourced from Invitrogen). Neural basal medium was supplemented with 20 ng/ml basic fibroblast growth factor (R&D Systems, Minneapolis, MN, USA) and 20 ng/ml epidermal growth factor (R&D Systems) prior to adding to the cells. Media was changed every two to three days, for a total culture period of 12 days at 37°C with 5% CO_2 _in a humidified incubator. Differentiated cells were fixed in 4% paraformaldehyde (BDH, Dorset, United Kingdom) for ten minutes at 4°C and immunostained overnight with 1 μg/ml of the mouse IgG_1 _anti-human β-tubulin III (Invitrogen); or 1 μg/ml mouse IgG_1_negative isotype control (Dako, Glostrup, Denmark). Cells were stained with 2 μg/ml of the goat anti-mouse Alexa568 or 488 IgG secondary antibody (Invitrogen) and counterstained with 0.5 μg/ml of 4, 6-diamidino-2-phenylindole (DAPI, Sigma) mounted in Vectashield (Vector Laboratories, Burlingame, CA, USA). Fluorescent images were captured as described below.

To test for epithelial differentiation capacity of the cells, cells were grown in Small Airway Growth Medium (SAGM, Lonza) as described by Berger *et al. *[55]. Cells were seeded at 5 × 10^4^/cm^2 ^in stem cell proliferation medium the day before the addition of SAGM and then were differentiated for eight days and medium was changed after four days. Differentiation was assessed by nested RT-PCR for Surfactant Protein C (SPC). RNA extraction and PCR protocols are detailed below. The PCR conditions for SPC were as described by Berger *et al. *[55], and the human bronchial epithelial cell line, 16HBE14o-, was used as a positive control for SPC expression [56].

### Reverse transcription-polymerase chain reaction

Total RNA was isolated from 5-10 × 10^5 ^trypsinized USSCs and from mechanically dislodged pools of spheres using RNeasy Mini Kit (Qiagen, Germantown, MD, USA) and RNase-Free DNase I Set (Qiagen) according to the manufacturer's instruction, and quantified using a NanoDrop 1000 Spectrophotometer. Reverse transcripts were prepared from 500 ng of total RNA using the SuperScript III First-Strand Synthesis System (Invitrogen), and cDNA was amplified using the Taq PCR Core Kit (Qiagen). Briefly, 0.4 μM of of forward and reverse primers, 0.25 U of Taq, 1× Q-solution, 1× PCR buffer containing 1.5 μM of MgCl_2_, 200 μM of dNTPs and 2 μl of cDNA were used. Table 1 contains the primer sequences, amplification conditions and expected RT-PCR product sizes of all genes investigated. The PCR conditions were as follows: 94°C for 2 minutes; 28-38 cycles of 94°C for 30 seconds, 30 seconds annealing at 51-61°C, 72°C for 1 minute; and a final extension step at 72°C for 2 minutes.

**Table 1:** Summary of conditions and primers used for RT-PCR.

Genes	Primer Sequences (5'-3')		Cycles	Size (bp)	Annealing Temperature (°C)
**Pluripotency-associated markers**					
*Oct4*	F	TCTCGCCCCCTCCAGGT	30	218	53
	R	GCCCCACTCCAACCTGG			
*Sox2*	F	GGCAGCTACAGCATGATGCAGGACC	34	130	60
	R	CTGGTCATGGAGTTGTACTGCAGG			
					
**Endodermal-associated markers**					
*FoxA2*	F	GGGAGCGGTGAAGATGGA	38	89	55
	R	TCATGTTGCTCACGGAGGAGTA			
*Gata6*	F	GAGTGAGAGAAGATGGAAGGG	38	250	55
	R	CGCACATGAAATCTGGATGTGG			
					
**Ectodermal-associated markers**					
*Nestin*	F	CAGCTGGCGCACCTCAAGATG	30	209	55
	R	AGGGAAGTTGGGCTCAGGACTGG			
*Pax6*	F	AACAGACACAGCCCTCACAAACA	30	275	55
	R	CGGGAACTTGAACTGGAACTGAC			
					
**Mesodermal-associated markers**					
*Brachyury*	F	GAACCAGCACCCTGTGTCCAC	38	608	57
	R	GCCACGACAAAAAGTCACTGC			
*Goosecoid*	F	CTCAACCAGCTGCACTGTC	38	322	55
	R	GTCAGCTGTCCGAGTCCAAATC			
					
**Control-associated markers**					
*GAPDH*	F	ATGGAGAAGGCTGGGGCTC	28	196	55
	R	AAGTTGTCATGGATGACCTTG			

### In situ hybridization

The spheres were fixed in 4% paraformaldehyde (BDH) for ten minutes at 4°C and incubated overnight in 20% sucrose in PBS with calcium and magnesium (Invitrogen) at 4°C. Cells were embedded in optimal cutting temperature compound (OCT, Tissue Tek, Japan) and stored at -70°C. Serial sections were cut (10 μm) using a Microm HM 550 cryostat (Carl Zeiss) and placed on Superfrost (Menzel-Glaser, GmbH, Fisher Scientific) slides. Haematoxylin and eosin (Sigma) staining was performed using standard conditions. To characterize the spheres, sections were stained with 4.5 μg/ml mouse anti-human Cytokeratin 8 IgG1κ (Invitrogen) and 2.5 μg/ml of mouse anti-human Oct4 IgG2β (Santa Cruz, CA, USA). Slides were washed in PBS (with calcium and magnesium) with 0.1% Tween20 (PBT), blocked for 2 hours with PBT containing 10% fetal calf serum (Invitrogen), and stained with the primary antibody or the appropriate IgG isotype control (Dako) overnight at 4°C. Cells were washed three times in PBT and stained with goat anti-mouse Alexa488 secondary antibody (Invitrogen) for 1 hour at room temperature and then washed three times in PBT. Before analysis, slides were counterstained with DAPI (Sigma) 0.5 μg/ml for five minutes at room temperature, then mounted with Vectashield (Vector Laboratories).

### Microscopy

Microscopy was performed on a fluorescent inverted microscope (Carl Zeiss AG, Oberkochen, Germany). Images were captured using Axiom photo system (Carl Zeiss).

## List of Abbreviations

bFGF: basic fibroblast growth factor; DAPI: 4',6-diamidino-2-phenylindole; hES cell: embryonic stem cell; Oct4: Octamer-4 homeodomain transcription factor; RT-PCR: reverse transcription PCR; and USSC: unrestricted somatic stem cell.

## Authors' contributions

FZ, RW, HA and PB conceived and designed the experiment. FZ and RW provided financial support. PB and DC developed the USSC growth medium^ACF^. FZ developed the USSCs lines. FZ, JK, MD and HA performed all the experiments. FZ, and PB performed all the data analysis. All authors were involved in data interpretation. All authors contributed to the manuscript writing and approved the final draft.
